# Draft Plastid and Mitochondrial Genome Sequences from Antarctic Alga *Prasiola crispa*

**DOI:** 10.1128/genomeA.01151-15

**Published:** 2015-10-08

**Authors:** Evelise Leis Carvalho, Gabriel da Luz Wallau, Darlene Lopes Rangel, Laís Ceschini Machado, Alexandre Freitas da Silva, Luiz Fernando Duarte da Silva, Pablo Echeverria Macedo, Antonio Batista Pereira, Filipe de Carvalho Victoria, Juliano Tomazzoni Boldo, Cháriston André Dal Belo, Paulo Marcos Pinto

**Affiliations:** a Applied Proteomics Laboratory, University of Pampa, São Gabriel, Brazil; b Antarctic Studies Plant Core, National Institute of Antarctic Science and Technology for Environmental Research, University of Pampa, São Gabriel, Brazil

## Abstract

The organelle genomes of the Antarctic alga *Prasiola crispa* (Lightfoot) Kützing have been sequenced. The plastid and mitochondrial genomes have a total length of 196,502 bp and 89,819 bp, respectively. These genomes have 19 putative photosynthesis-related genes and 17 oxidative metabolism-related genes, respectively.

## GENOME ANNOUNCEMENT

Antarctica has attracted considerable interest from biologists interested in understanding the evolutionary adaptation of extremophile organisms (1). *Prasiola* spp. are the best-known Antarctic algae found at many terrestrial and supralittoral sites, representing the most important primary producers (1–3). The species most commonly reported is *Prasiola crispa* (Lightfoot) Kützing. *P. crispa* has several interesting biological features: it typically grows on moist soils fertilized by penguin guano (4), tolerates repeated thaw cycles in the spring and fall and freezing over winter, and absorbs high levels of UV radiation during summer (5).

The organelle DNA was sequenced by Macrogen Service in a Solexa-Illumina HiSeq 2500 next-generation sequencing device according to the manufacturer’s instructions. A paired-end approach with a read size of ~100 bp was employed. Sequence assembly was performed with SOAPdenovo2 software version 2.01 (6). All open reading frames were annotated using CpGAVAS (7) and Mitofy (8) for chloroplast and mitochondrial genomes, respectively.

The *P. crispa* plastid genome (cpDNA) information resides on a single molecule with a total length of 196,502 bp and a G+C content of 29.32%. Compared with others species from the *Prasiola* clade, *P. crispa* are among the largest (*Prasiolopsis* sp., 306.1 kb; *Pabia signiensis* T. Friedl and O’Kelly, 236.5 kb; *Koliella longiseta* (Vischer) Hindák, 197.1 kb; and *Stichococcus bacillaris* Nägeli, 116.9 kb) (9), even when compared to other species close to *Prasiolales*, such as *Chlorella mirabilis* V. M. Andreyeva (168.0 kb) (9). The cpDNA comprises 63 putative coding genes, 26 tRNAs, and 2 rRNAs. Among those, we were able to annotate at least 19 putative protein-coding genes related to photosynthesis, such as photosystem I and II putative proteins.

The *P. crispa* mitochondrial genome (mtDNA) is the first mitochondrial sequenced genome from the *Prasiolales* order. It has a total of 89,819 bp and a G+C content of 29.29%, and compared with others species from the *Trebouxiophyceae* class, *P. crispa* has the largest mtDNA (*Trebouxiophyceae* sp., 74.4 kb; *Chlorella* sp. ArM0029B, 65.0 kb; *Oltmannsiellopsis viridis* (P. E. Hargraves and R. L. Steele) M. Chihara and I. Inouye, 56.8 kb; *Prototheca wickerhamii* Tubaki and Soneda, 55.3 kb; and *Chlorella sorokiniana* Shihira and R. W. Krauss, 52.5 kb) (10–14). The *P. crispa* mtDNA genome has 56 genes, comprising 32 putative protein-coding genes, 21 tRNAs, and 3 rRNAs. Among those, we were able to annotate at least 17 protein-coding genes related to mitochondrial oxidative metabolism, such as mitochondrial respiratory chain complex I, III, and IV putative proteins.

These organelle genomes are the first draft genomic sequences obtained from Antarctic *Trebouxiophyceae* algae. Mitochondria and chloroplast organelles play a pivotal role in energy metabolism and are great tools for taxonomic analysis in higher plants and algae. The plastid and mitochondrial genome data would be useful for further genetics studies, phylogenetic analysis, and resource protection of *P. crispa* and phylogenetic analysis of *Trebouxiophyceae* green algae.

### Nucleotide sequence accession numbers.

The cpDNA and mtDNA contig sequences were deposited in GenBank under accession numbers, KR017748, KR017749, and KR017750, and KR017746, and KR017747.
